# Phosphorus Effects of Mesoporous Bioactive Glass on Occlude Exposed Dentin

**DOI:** 10.3390/ma6115335

**Published:** 2013-11-19

**Authors:** Wen-Cheng Chen, Cheng-Hwei Chen, Jung-Chang Kung, Yu-Cheng Hsiao, Chi-Jen Shih, Chi-Sheng Chien

**Affiliations:** 1 Advanced Medical Devices and Composites Laboratory, Department of Fiber and Composite Materials, College of Engineering, Feng Chia University, Taichung 40724, Taiwan; E-Mail: wencchen@fcu.edu.tw; 2 School of Dentistry, College of Dental Medicine, Kaohsiung Medical University, Kaohsiung 80708, Taiwan; E-Mail: endojhchen@gmail.com; 3 Department of Family Dentistry, Kaohsiung Medical University Hospital, Kaohsiung Medical University, Kaohsiung 80708, Taiwan; E-Mail: den920332@gmail.com; 4 Department of Fragrance and Cosmetic Science, Kaohsiung Medical University, Kaohsiung 80708, Taiwan; E-Mail: dentinychsiao@gmail.com; 5 Department of Fragrance and Cosmetic Science, Kaohsiung Medical University, Kaohsiung 80708, Taiwan; 6 Department of Orthopaedics, Chi Mei Foundation Hospital, Tainan 71004, Taiwan; 7 Department of Electrical Engineering, Southern Taiwan University of Science and Technology, Tainan 71005, Taiwan

**Keywords:** mesoporous bioactive glass, phosphoric acid, dentin hypersensitivity

## Abstract

In recent studies, sealing of exposed dentinal tubules is generally considered as one of the most effective strategies to treat dentin hypersensitivity. Mesoporous bioactive glass (MBG) is a potential material for treating dentin hypersensitivity due to its highly specific areas for dissolution and re-precipitated reaction for reduction in dentin permeability. The groups of commercial products of PerioGlas^®^, synthetic MBG and MBG without phosphorus (MBGNP) were compared. The MBG and MBGNP powders were prepared by the sol-gel method and mixed with different calculated ratios of phosphoric acid (PA) and then was brushed onto dentin surfaces. We used X-ray diffractometer (XRD), scanning electronic microscope (SEM), and Fourier transform infrared spectroscopy (FTIR) to investigate the physiochemistry and the occlusion ability of dentinal tubules. The results showed that MBG paste mixed with PA solution has a better ability for occluding dentinal tubules than MBGNP; it has a short reaction time and good operability. The major crystallite phase of MBG agents was monocalcium phosphate monohydrate [Ca(H_2_PO_4_)_2_·H_2_O] in the early stages of the reactions. MBG pastes that were mixed with 30% and 40% PA had the ability to create excellent penetration depth greater than 80 μm. These agents have the potential to treat dentin hypersensitivity.

## 1. Introduction

The occurrence of dentin hypersensitivity is clinically prevalent. Most patients develop the sensation of sudden, intense sharp pain during ingestion of ice cold liquids, acidic food, sweets, while brushing, or even when flossing. The main causes for dentin hypersensitivity are dentin exposure, mostly of the canine and premolar areas [1]. According to statistics, between 14 and 30 percent of adults suffer from dentin hypersensitivity and this proportion increases with age [2,3,4]. Therefore, there is a huge market to develop a dentin desensitizing paste. However, there are major challenges. Holland *et al.* [5] defined dentin hypersensitivity as the short but sharp sensation during different external stimuli to the exposed dentin, which cannot be attributed to any other form of dental defect or disease. These external stimuli can be categorized into chemical, physical, mechanical, or pathological stimuli which all contribute to exposing dentinal tubules. Dentinal tubules are thin at the surface (0.6–0.8 μm) and thicker near the dental pulp (up to 3 μm) [6]. Every dentinal tubule is surrounded by a transparent circular strip making up the tubule wall, referred to as the peritubular dentin. This layer of structure is deficient of collagen and mineralizes easily, forming a structure mainly composed of carbonate hydroxyapatite and hardness five times the intertubular dentin. When dentin is 3 mm thick, external stimuli such as those mentioned previously do not easily penetrate and stimulate the pulp cavity; however, once the dentin is reduced to only 0.3 mm, external stimuli easily reach the pulp cavity [7].

The treatment for dentin hypersensitivity consists of primarily chemical desensitization and physical desensitization methods.

The chemical hypersensitivity elimination method usually involves protein precipitation, where chemicals are used to precipitate proteins within the dentin tubules to reduce fluid disturbance. Although this successfully reduces hypersensitivity, the practice of protein precipitation is gradually being phased out as it induces a permanent color deposition on the surface of the teeth and presents a stimulus to the surrounding gum and pulp [8]. An alternate method to reduce hypersensitivity is tubule occlusion, described as using chemicals to seal the dentin tubules by forming crystals or minerals to reduce or seal the opening end of the dentin tubules. Currently, most procedures to treat hypersensitivity involve tubule occlusion [9,10,11,12,13].

Recently, mesoporous bioactive glass (MBG) has been introduced as a restorative material for bone implantation. It is developed using a mesoporous material processing technique with pores ranging from 2 to 50 nm. This technique allows for high specific surface area of the mesoporous structure to be produced with the capability to carry drugs. Currently, different types of bioactive glass (BG) have been applied in dental science literature as well as dentin tubules occlusion [14,15,16]. The main ingredient of bioactive glass (BG) is SiO_2_–CaO–Na_2_O–P_2_O_5_. In the past, BG has been used to maintain ridge shape and integrity after teeth extraction in dentistry as an endosseous ridge maintenance implant, which is mainly composed of 45% SiO_2_–24.5% CaO–24.5% Na_2_O–6% P_2_O_5_ (wt %) [17,18]. In addition, scholars have proposed that when BG powder contacts water, sodium ions are released and increase the pH to a base. Under these circumstances, the calcium ions and phosphate ions that infiltrate dentinal tubules form apatite to occlude the dentin [19,20]. However, studies involving MBG in the treatment of dental illness are sparse. Our study also showed that BG with mesoporous structures turned the pastes mixed with suitable phosphoric acid solution into a material with great ability for occluding dentinal tubules; it has a short reaction time and good operability, and these agents have better potential for the treatment of dentin hypersensitivity than BG without mesoporous structures [21]. Furthermore, this study outlines the use of BG prepared without phosphor as a control group to compare the effects of phosphate ions and mesoporous structure on tubule occlusion.

## 2. Results and Discussion

### 2.1. The Setting Properties of Commercialized Product PerioGlas^®^

**Figure 1:**
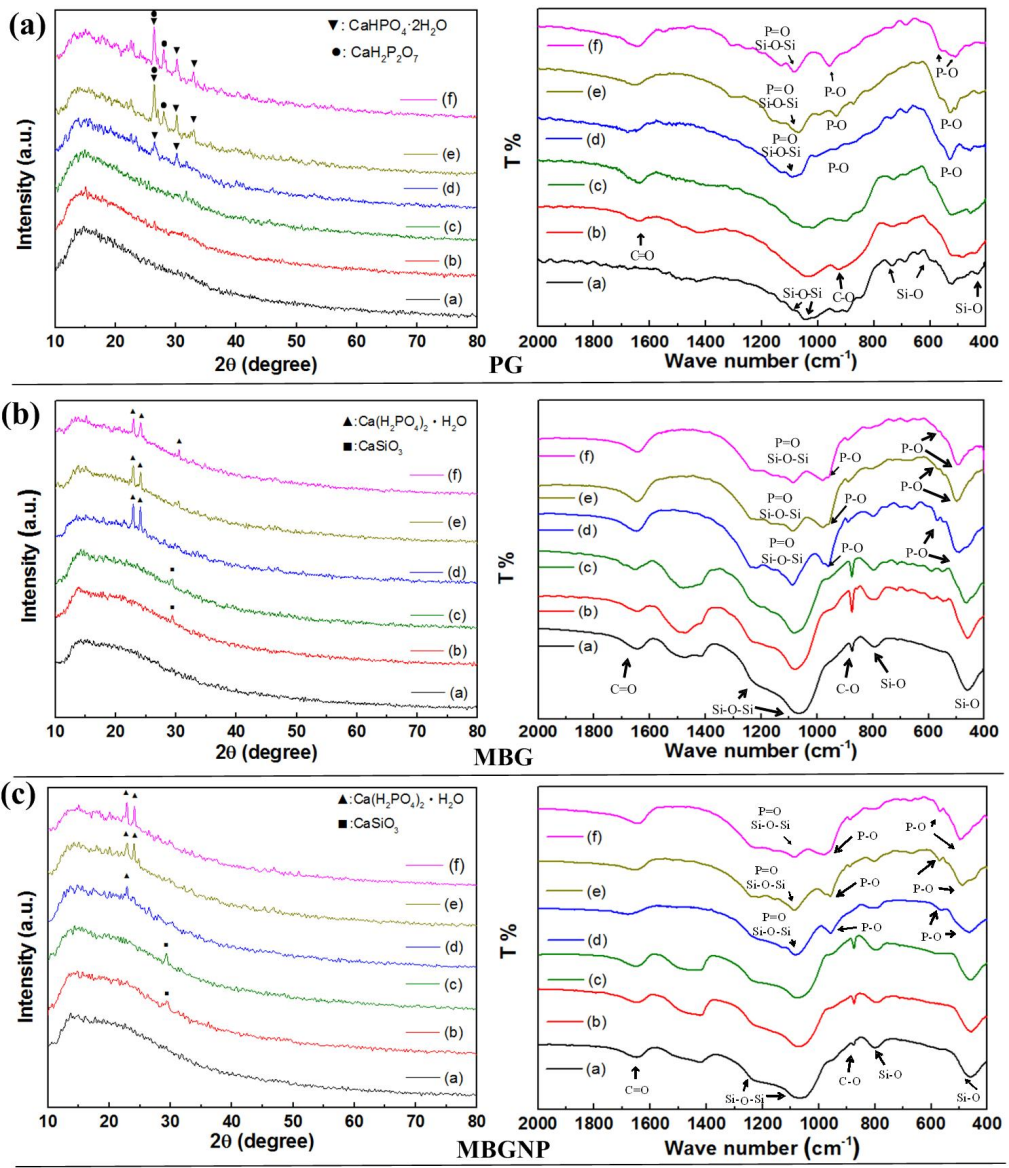
The X-ray diffractometer (XRD) pattern and Fourier transform infrared spectroscopy (FTIR) spectra of (**a**) PerioGlas (PG), (**b**) mesoporous bioactive glass (MBG), and (**c**) MBG without phosphorous (MBGNP) mixed with varied luting solutions for 10 min. The condition labels in figure are: (**a**) PG; (**b**) PG/W; (**c**) PG/PBS; (**d**) PG/20PA; (**e**) PG/30PA; (**f**) PG/40PA (the symbol of PG/ indicate the PG reacted with varied solutions: W: de-ionized water; PBS: phosphate buffered saline; 20, 30 and 40PA: 20, 30 and 40 wt % PA solution).

The pastes of PerioGlas (PG) mixed with de-ionized water and PBS had nearly the same XRD patterns with the original PG powders after 10 min of reaction (Figure 1a). The products of the PG pastes mixed with varied concentrations of PA were biphasic, consisting of CaHPO_4_·2H_2_O and CaH_2_P_2_O_7_ after reaction. The FTIR spectra show a peak at 530 cm^−1^ identified as P–O. Furthermore, there is a broad peak at 1000–1100 cm^−1^, representing double absorption bands of Si–O–Si asymmetric vibration, overlapping with P–O. The peak at 852 cm^−1^ is absorption bands of symmetric Si–O stretch vibration and the peak at 1650 cm^−1^ is C=O (Figure 1a). The pastes of PG mixed with de-ionized water and PBS had no obvious difference in the functional spectra compared to the original PG powders after 10 min of reactions. When PG was mixed with phosphorus, PG/20PA, PG/30PA, and PG/40PA showed peaks in the 530 and 570 cm^−1^wavelengths as P–O as well as 960 cm^−1^ as P–O–P.

### 2.2. Effects of Commercialized PerioGlas^®^ Brushed onto Dentin-Occlusion and Penetration in the Depths of Dentinal Tubules

**Figure 2:**
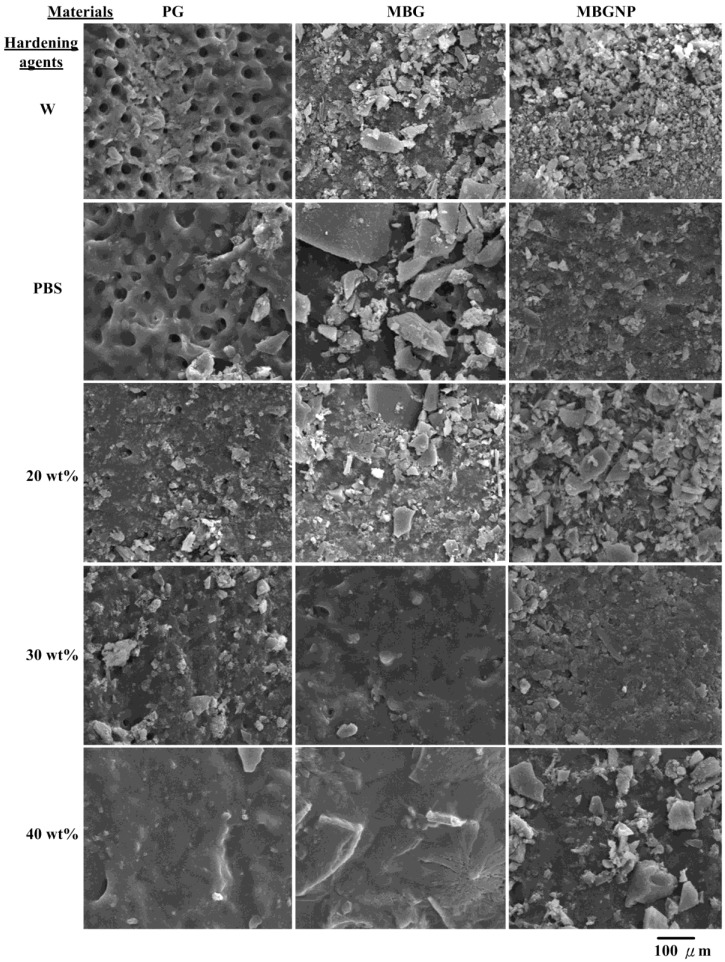
SEM micrographs of dentin specimens for topographies of different materials of PG, MBG and MBGNP reacted with various hardening solutions for 5 min at a magnification of 1000×. (W: de-ionized water; 20PA, 30PA and 40 PA: respective 20, 30 and 40 wt % PA).

**Figure 3:**
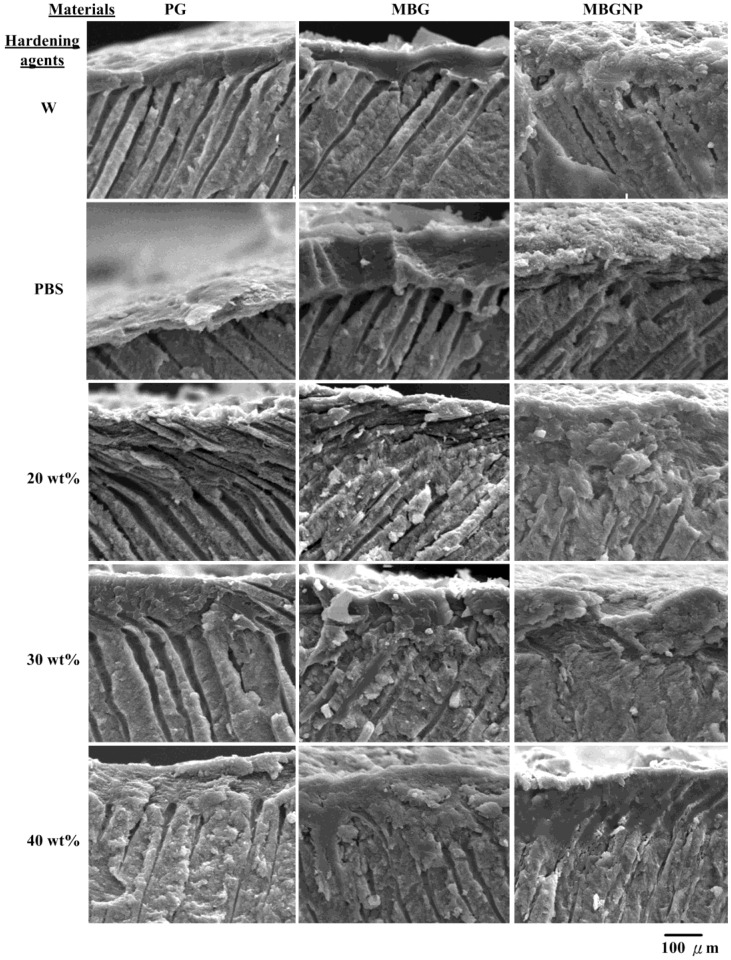
SEM micrographs of dentinal tubules cross-sectional morphologies treated with different materials of PG, MBG and MBGNP reacted with various hardening solutions for 5 min at a magnification of 1000×. (W: de-ionized water; 20PA, 30PA and 40PA: respective 20, 30 and 40 wt % PA).

The paste of PG reacted with de-ionized water (PG/W) was brushed onto the dentinal surfaces and the results showed that the paste is not efficient in sealing dentinal tubules (Figure 2). The dentinal tubules sealed less than one-third of the tubules while the thickness of the protected layers on the dentinal topographies were less than 100 μm. The PG paste that reacted with PBS (PG/PBS) is much better at sealing than PG/W, although some tubules were still open. The inside of the dentinal tubules had no precipitation and exhibited no occlusion of dentinal tubules, although not all pores were sealed by the pastes. The occlusion efficiency onto dentinal tubules in the group of PG mixed with 20 wt % PA (PG/20PA) was enhanced, but most of the interior of the tubules still had little or no precipitates. At most, precipitates were only formed in 13% of the tubules, and the permeable depths of the precipitates in the dentinal tubules were approximately 68 μm on average (Table 1 and Figure 3). The surface occlusion efficiency of PG/30PA is higher than PG/20PA. Not only were dentinal tubule openings sealed, most surface crystalline precipitates were also merged together into the tubules, reaching up to 70 μm in depth. Nonetheless, the precipitates only filled 13% of the tubules as well (Table 1). On the other hand, PG/40PA surfaces were solid and smooth. The paste formed a crystal layer which was superior to the other groups. While the surface closure was extremely good, the dentinal tubules still did not contain any precipitates. This can be attributed to the low ion release rate after PA and phosphates were mixed together. Although there is a high crystallization rate, the paste is unable to flow smoothly after forming a slurry dynamic, causing blockage at the surface of the powders. In the case of 20% and 30% phosphorus containing powders, the amount of phosphate ions is less than PG40PA, and it is this low ion concentration that allows the slurry paste to flow at a higher liquidity. This in turn allows for a longer time for dissolution-precipitation reactions into the dentinal tubules, resulting in the small amounts of observed crystalline precipitates. Commercialized product of PG mixed with PA solution only had the partial ability to occlude dentinal tubules with only partial crystalline precipitates in the interior. As such, when the surface erodes due to external stimuli, the inside becomes exposed to further external stimuli and may give rise to dentin hypersensitivity again.

**Table 1:** The percentage of tubule occlusion and the penetration depth of commercialized PG, synthetic MBG and MBG without phosphorus (MBGNP) based occlusive agents for 10 min mixing with hardening solution (*n* = 20).

Occlusive agents mixed with varied hardening agents	PG			MBG			MBGNP		
Concentrations of phosphate (wt %)	20PA	30PA	40PA	20PA	30PA	40PA	20PA	30PA	40PA
Percentage of tubule occlusion (%)	13%	13%	0 ^a^	50%	68%	65%	0%	0%	25%
Average of penetration depth (μm)	68.2	74.1	0	68.4	71.5	73.1	0	0	50.8
Standard deviation of penetration depth (μm)	2.0	2.1	0	9.2	11.1	9.4	0	0	7.2

^a^ indicated the materials existed no efficiency to occlude and penetrate.

### 2.3. The Setting Properties of Meso-Porous Bioactive Glass (MBG) with Phosphate

**Figure 4:**
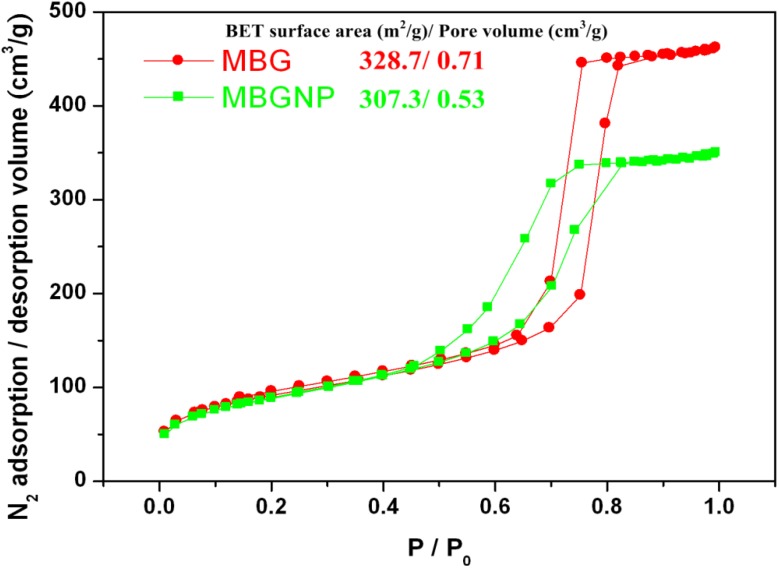
Nitrogen adsorption/desorption isotherm and the Brunauer–Emmett–Teller (BET) surface area with pore volume of MBG and MBGNP.

As shown in Figure 1b, the MBG groups mixed with de-ionized water and PBS showed a new product phase of CaSiO_3_ (29.4°) in the XRD patterns and had not significant differences in crystal phases. The mixing of PA solutions with MBG revealed two peaks, 22.9° and 24.1°, signifying monocalcium phosphate monohydrate [Ca(H_2_PO_4_)_2_·H_2_O, MCPM] with the triclinic crystal structure [16] (Figure 4).

Under FTIR analysis, the spectrum in Figure 1b showed that where the peak originally existed at 1650 cm^−1^ as the C=O asymmetric stretching bands. Furthermore, there is the 875 cm^−1^ C–O group asymmetric bending vibration and the 1000–1250 cm^−1^ Si–O–Si asymmetric stretching band. The 800 cm^−1^ represents the Si–O–Si symmetric stretching band while the 475 cm^−1^ band is the Si–O bending vibration band. The difference in MBG/W and MBG/PBS is more subtle. Similarly, the peaks were made up of silicate and carbonate based functional groups. The samples containing phosphorus, MBG/20PA, MBG/30PA, and MBG/40PA, showed a few extra peaks compared to MBG. These peaks included the 970–1130 cm^−1^ P=O band that stretches asymmetrically, the Si–O–Si asymmetrical stretching and vibration double absorbance, the 960 cm^−1^ peak of P–O stretching vibration, and finally the 570 cm^−1^ peak from the P–O symmetrical stretching vibrational absorption.

### 2.4. Effects of MBG with Phosphorus on Penetration Depth of Dentinal Tubule

Compared to PG groups, MBG/W and MBG/PBS (Figure 2) decreases surface pores more, although there is still a lack of crystalline precipitates within dentinal tubules. The surface of MBG/20PA group is covered by uneven sizes of crystals, while below the surface, there are effects of apparent crystallite fusion (Figure 2). While crystal particles can be seen, dentinal tubules were no longer exposed from the surface with the remaining exposed dentinal tubule diameter decreased to around 1 μm and the depth to about 68 μm at a 50% occlusion rate (Table 1). In contrast, MBG/30PA and MBG/40PA results were vastly different from the MBG/20PA group. Apart from crystallization, the surfaces have formed a homogenous, smooth, crystallite fusion in the smear layers. These results suggest that under the 30 and 40 wt % of phosphate-containing hardening/lubricating solution, the ions in the crystallites formed from MBG have an enhanced diffusion rate. The faster diffusion rate formed a dense crystallite fusion layer penetrating depths of 71.5 and 73 μm, respectively. The occlusion rates were 68% and 65%, respectively, for MBG/30PA and MBG/40PA with respective smeared surface layers of 15 and 20 μm (Figure 3). Furthermore, results show that the phosphate mixture does in fact have the abilty to enter dentinal tubules with both MBG30PA and MBG40PA samples showing excellent occlusion effects without any statistical significance between each other.

### 2.5. The Setting Properties of MBG without Phosphorus (MBGNP)

XRD analysis in Figure 2c shows that the MBGNP/W and MBGNP/PBS patterns are similar to before mixing, resembling the MBG group. At 29.4°, there is a peak indicating the presence of CaSiO_3_ crystals. MBGNP/30PA and MBGNP/40PA patterns show MCPM crystal phase at 22.9° and 24.1° whereby MBGNP/20PA contains a small diffraction peaks appearance at 22.9°. FTIR spectra of original powders of MBGNP, MBG, and reactions of MBGNP, MBG/W and MBGNP, MBG/PBS are similar. Most functional groups discovered were silicon-oxygen bond or carbonate based. However, compared to MBG, patterns from MBGNP/20, 30, and 40PA groups reveal extra characteristics including 970–1130 cm^−1^ P=O asymmetric stretching bands, 960 cm^−1^ P–O caused by stretching vibration, and 570 cm^−1^ P–O caused by symmetric bending vibration. The bonding spectra of the FTIR are basically similar. The difference between MBGNP and MBG are mainly the 30.5° diffraction peak due to the absence of phosphoric acid, decreasing the yield of crystalline products in MBGNP20PA. This result is also observed by SEM.

### 2.6. Effects of MBG without Phosphorus (MBGNP) on Penetrate in the Depth of Dentinal Tubule

In the MBGNP group, MBGNP/W, PBS occluded the surface of dentinal tubules similarly to MBG/W, PBS. However, the surface crystals were finer in morphology compared to MBG/W, exposing larger dentinal tubules (Figure 2). The respective opening diameters were 1.5–2.5 μm and 1.5–2.0 μm for MBGNP/W, PBS. The groups of MBGNP/W, PBS had the same results with MBG groups, which showed no penetration of precipitations into dentinal tubules (Figure 3). Of particular note, the group of MBGNP/20PA substrate surface contains crystals of varying size, exposing dentinal tubule openings 0.5–1.5 μm without any crystalline precipitates inside. Although a portion of MBGNP/30PA have fused together on the topographies of dentinal tubules, the exposed tubules were 0.5–1 μm. However, crystalline precipitates were also absent from the inside of dentinal tubules. The surfaces of MBGNP/40PA already have a large extent of crystalline fusion where granular crystals also fused together. Furthermore, crystalline precipitates can also be found inside dentinal tubules reaching depths of 50.8 μm and an occluding rate of 25% (Figure 3 and Table 1).

### 2.7. Nitrogen Adsorption-Desorption Texture Properties of MBG and MBGNP Mixed with 30 wt % Phosphoric Acid

**Figure 5:**
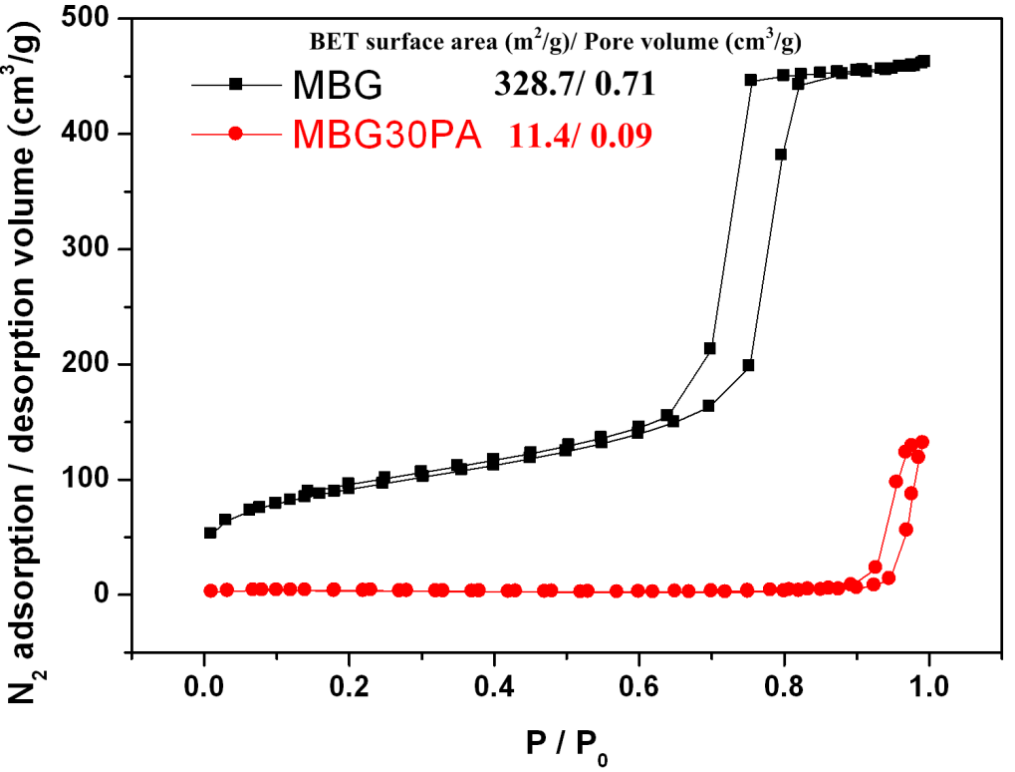
Nitrogen adsorption/desorption isotherm and the BET surface area with pore volume of MBG before and after mixed with 30% PA.

It shows a comparison of the specific surface areas and mesoporous volumes were determined by the nitrogen adsorption and desorption isotherms, which are depicted in Figure 4. The curves for MBG and MBGNP are identified as a type-IV hysteresis loop, which is associated with meso-meter pores. The curves for MBG and MBGNP are typical type-H1 hysteresis loops of one-dimensional channels [22]. The specific surface areas are also as shown in the Figure 5 and that shows the disappearance of mesoporous structures in MBG samples after being mixed with 30% PA. Comparing the specimen of MBG/30PA to original powders of MBG shows that the specific surface area and mesoporous volume significantly decreased to 4% and 12.6%, respectively.

## 3. Discussion

### 3.1. Commercialized PG and Synthesized MBG

The mixing of PG with varying concentrations of PA for 10 min resulted in a mixture of dicalcium phosphate dihydrate (DCPD) and MBG with a different solubility compared to MCPM (Figure 1). Because DCPD is a precursor to hydroxyapatite (HA), a metabolic phase of octacalcium phosphate (OCP) precipitate easily forms. During reaction, the pH value is also higher than MCPM. This results in a higher viscosity of the product, limiting the initial reaction product fluid dynamics of the slurries and decreasing the ability to flow smoothly into the dentinal tubules [15].

### 3.2. Synthesized MBG with and without Element of Phosphorus

The groups of MBGNP involved crystals with smaller sizes than those found in the MBG group. This observation suggests that the MBGNP reaction rate is slower than that of MBG. With the addition of PA, MBGNP/20, 30PA substrate surfaces formed large amounts of crystalline precipitates to successfully seal the dentinal tubules (Figure 2). However, crystalline precipitates were absent from the inside of the tubules. Although MBGNP/40PA surfaces were completely covered, surface granular crystallites may have resulted from newly formed crystals or from upper level crystals not infused to the bottom crystallization. Among MBGNP groups that reacted with varying concentration of phosphate, only the MBGNP/40PA substrate contains crystalline precipitates within the dentinal tubules. On the other hand, the MBG formula formed crystals in the MBG/20PA group to a depth of 68.4 μm with an occlusion rate of about 50%, double of MBGNP40PA (Figure 3 and Table 1). The above results suggest that MBG does not carry an abundant of phosphate ions. However, MBG does contain a decisive ability to allow phosphate ions to infiltrate dentinal tubules.

### 3.3. Textural Properties of MBG and MBGNP Reacted with 30 wt % Phosphoric acid

Figure 4 showed that MBGNP specific surface area and dentinal tubule opening diameters have both decreased compared to MBG. Specifically, tubule opening decreased by 25% in terms of diameter. Because MBG is structured with SiO_4_ in a mesh network as the mainframe while CaO and P_2_O_5_ are connected to this mainframe, the P_2_O_5_ functional group is missing. As a result, steric hindrance is limited and decreases the mesoporous volume. The decrease in the mesoporous volume leads to a lower in reaction space, thus limiting the release of ions. Consequently, the ability of the MBGNP paste to form crystalline precipitates decreases.

The reasons specific surface area and pore volume of MBG/30PA obviously rather decreased relatively to MBG (Figure 5) was due to the following. First, MBG mixed with PA released calcium ions while SiO_4_ dissociates into ionic states, which disrupts the mesoporous structure and decreases both specific surface area and mesoporous volume. Second, the released calcium ions reacts with phosphate ions from PA to form calcium dyhydrogen phosphate crystals on the surface of dentin or MBG particles, of which the latter also decreases the specific surface area and mesoporous volume.

### 3.4. Summarized Discussions

The study discovered that three days after PA and dentin substrates have reacted, crystalline precipitates can be found inside the dentinal tubules [23]. It is proposed that the crystals could have directly come from the reaction between PA and dentin, which is a result of the peritubular dentin releasing calcium phosphate ions that form precipitates. On the other hand, this study confirms that MBG combined with deionized water and PBS reacts only to form precipitates on the surface of dentin substrates. These precipitates are unable to enter dentinal tubules to form crystalline precipitates. Thus, to form crystalline precipitates within the dentinal tubules, an acidic environment is crucial.

Clinical studies have shown that smear layers commonly exists. Whether using a manual or a mechanical rotary shaving device, the shaving of dentin turns the hard tissue into pulverized powder and fragments consists of mainly minerals and collagen fibers along with hydroxyapatite, bacteria, blood, and dead tissue [24]. The smear layer can be divided into two sub-layers. First, the smear is spread on the surface of the dentin at a thickness of 2–5 μm. Second, the particles or pulverized powder enter dentinal tubules to form smear plugs. Generally speaking, larger oral cavity dentinal tubule openings will form larger and deeper smear plugs. After root canal treatment, smear plugs may reach a depth of 40 μm [25]. Smear layers form to reduce hydraulic conductance [26] and dentin sensitivity [27].

However, Pashley conducted a test targeted towards combining different materials and smear layers and discovered a fracture surface between the material and the smear layer. According to these results, the binding force between the smear layer and the dentin is around 3–5 MPa [25]. The existence of the smear layer and the smear bolt will interfere with the material and the dentin to contact or enter the dentinal tubules [28]. Thus, if the binding between the material and the dentin is to be increased, the smear layer must be eliminated [29]. Clinically, there are many methods to remove the smear layer. One of the most effective involves using 37% PA to expose the dentinal tubules completely [29]. The use of acid etching to remove the smear layer is because PA was thought to cause pulp inflammation [30,31,32]. However, this was later attributed to the smear layer being removed and exposing the dentin surface to external stimuli and causing pulp disease complications [33,34]. Currently, dentin itself creates hydroxyl ions to neutralize the effects of acid [30].

Although the acid environment plays an important role in the formation of crystalline precipitates, PA should not be used alone. The dentin substrate deteriorates and falls apart during prolonged exposure to PA. This results in calcium loss and the substrate distorts easily. From the study, PA mixed with mesoporous bioactive glass will protect the dentin substrate surface by providing a layer of crystallite fusion. Therefore, it is speculated that the mesoporous bioactive glass limits PA invasion of the dentin, thereby protecting it.

### 3.5. Research Limitations

The teeth used throughout the study were molars. Since different teeth have different shapes and forms, including the number of dentinal tubules, further analysis using different teeth will be required. Specifically, the ability of MBG to occlude in different models will need to be studied. Furthermore, the oral cavity is often subjected to external damages, such as ingestion, teeth brushing, *etc.* A future study could incorporate material fatigue and wear testing to observe the effects on smear layer after successive wear simulations. The effects on thickness of the crystal or peeling of the smear layers will be observed to test whether dental tubules are exposed.

## 4. Experimental Section

### 4.1. Preparation of Materials

The materials used in this study can be referred to in Table 2.

**Table 2:** Sources of materials.

Materials	formula	Company	Purity (%)
Pluronic F-127	EO_106_–PO_70_–EO_106_	BASF	–
Polyurethane foam	–	–	–
Calcium nitrate tetrahydrate	Ca(NO_3_)_2_·4H_2_O	SHOWA	98.5
Triethyl phosphate	C_6_H_15_O_4_P	HANAWA	98.0
Tetraethyl orthosilicate	C_8_H_20_O_4_Si	ACROS	98.0
Hydrochloric acid	HCl	Riedel-de Haen	98.0
Ethanol	C_2_H_5_OH	J. T. Baker	99.9
Phosphoric acid	H_3_PO_4_	SHIMAKYU	98.0

#### 4.1.1. Compare groups of Commercialized Product

The control group of commercialized PerioGlas**^®^** (Bioglass^®^ Synthetic graft Particulate) is used as a bioglass control throughout the study. The main component ratios are SiO_2_ 45.0%, Na_2_O 24.5%, CaO 24.5%, P_2_O_5_ 6.0%.

#### 4.1.2. Powder Preparation of Meso-Pore Bioglass with Phosphate

In a typical synthesis, F127 (7.0 g), tetraethyl orthosilicate (TEOS, 6.7 g), Ca(NO_3_)_2_·4H_2_O (1.4 g), triethyl phosphate (TEP, 0.73 g; 80:15:5 Si:Ca:P molar ratio), and 0.5 M Hydrochloric acid ( HCl, 1.0 g, Riedel-de Haën, Sigma-Aldrich, St. Louis, MI, USA) were dissolved in ethanol (60 g) and stirred at room temperature for 1 day [35]. After this, polyurethane foam was completely immersed in the sol-gel solution and compressed, to force the sol-gel to migrate into the pores of the foam. Excess sol-gel was then squeezed out. The struts of the sponge body tissue were uniformly coated with the appropriate sol while the pores remained open. After 24 h of drying, the procedure was repeated. The raw porous scaffold bodies were dried for several days in open air at room temperature and then dried at 100 °C for 24 h. After the samples were completely dry, they were thermally treated at constant heating rates (10 °C/min) to calcination temperatures in the range of 600 °C, and then left at these temperatures for 2 h. This stage was designed to decompose and eliminate the surfactant F127 and the polyurethane foam support without collapsing the mesopores and macropores. After heating and cooling, the powders were washed three times with acetone and dried in open air. They were then subjected to characterization and analysis.

#### 4.1.3. Powder Preparation of Meso-Pore Bioglass without Phosphate 

In a typical synthesis, F127 (7.0 g), tetraethyl orthosilicate (TEOS, 6.7 g), Ca(NO_3_)_2_·4H_2_O (1.4 g), (80:15 of Si:Ca molar ratio), and 0.5 M HCl (1.0 g) were dissolved in ethanol (60 g) and stirred at room temperature for 1 day. To form the powders, the sol-gel solution was processed in the same procedure as described above.

### 4.2. Remineralized Ability Evaluation to Penetrate into the Depth of Dentinal Tubule

Treatments of commercial products of PerioGlas^®^ powders were used as the control group, namely PG. Two experimental groups were studied, namely silicate bioactive glass containing phosphate (MBG) and bioactive glass without phosphate (MBGNP). The three powders were mixed with the luting/hardening solutions of de-ionized water, phosphate buffered saline (PBS), 20, 30 and 40 wt % PA solution.

**Figure 6:**
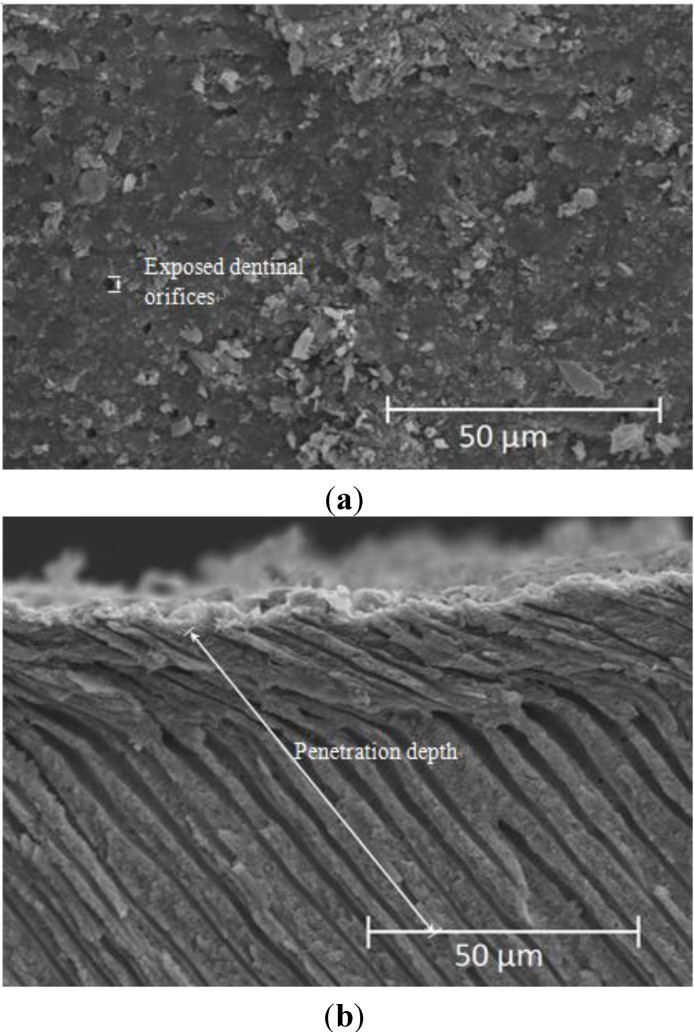
Examples of SEM mapping the occlusion calculated for the (**a**) occlusion rate and (**b**) penetration depth.

The preparation method of the dentin samples has been presented in our previous study [21]. The dentin samples were prepared from the caries-free human molars extracted for surgical reasons from healthy patients. Teeth were obtained after approval by the Institutional Review Board of Kaohsiung Medical University Hospital, Kaohsiung, Taiwan. The upper dentin surfaces of each 1 mm thick sample were sand published with 800-grit SiC paper for 1 min and followed by ultrasonic cleaning for 10 min as a standard flat dentin surface with opened dentinal tubes. The produced paste were spread on the dentin surface samples and incubated at 37 °C at 100% relative humidity, to simulate natural environment of the oral cavity. Occlusion times were set at 10 min and were immediately removed and washed with large quantity of deionized water for 20 s and submerged in anhydrous ethanol to stop any reactions. After being dried for 24 h, the samples were mechanically split open for analysis of osmosis efficiency. The inside cavity is plated with gold and the degree of occlusion and crystallization in the dentinal tubules of each group were compared. The occlusion rate and penetration depth of the dentin tubules are calculated as from SEM images. Twenty dentinal tubules from three different sets of SEM images were used for sampling. The occlusion rate is defined as the ratio of dentinal tubules with crystallization to total number of dentinal tubules. The penetration depth is defined as the average length of the crystallization of all the dentinal tubules in μm (Figure 6).

### 4.3. Textural Characterization

X-ray diffraction (XRD, Rigaku D-max IIIV, Tokyo, Japan) at a scanning speed of 4 /min within the 2 theta range of 10°–80° and Fourier-transform infrared spectroscopy (FTIR) analyses (Thermo NICOLET 6700, MA, US) were performed. To study the re-mineralized topographies and the composition of elements on cross-section topographies, the samples were examined using a field emission scanning electron microscope (SEM) (Hitachi S-3000N, Hitachi, Tokyo, Japan) equipped with an energy dispersive X-ray spectrometer (EDS, Horiba EX220, Tokyo, Japan). Nitrogen adsorption and desorption isotherms were measured at 77 K on a Quantachrome Autosorb 1 sorption analyzer. All samples were outgassed for 12 h at 150 °C under high vacuum in the degas port of the adsorption analyzer. The specific surface areas of the samples were measured using the BET method (ASAP 2010, Micromeritics, Norcross, GA, USA) [36] with nitrogen as an absorbent.

### 4.4. Statistical Analyses

The statistical analyses of the results used one-way ANOVA to investigate the significant group comparisons between different populations using the JMP 9.0 software (SAS Institute, Inc., Cary, NC, USA). In all cases, the results were considered to be significantly different when *p* < 0.05.

## 5. Conclusions

The formed precipitation of dicalcium phosphate dihydrate with a depth of 60–80 micrometers proved that the MBG exhibited an excellent reduction in dentin permeability when the MBG paste was mixed with PA solution. Phosphate concentrations of 30% and 40% resulting MBG products yielded the best homogenous smear layers that were smooth with well fused typographies. This allowed the surface crystallite precipitates to stay firm and not peel off in the event of an external force.

MBG is superior in both occlusion and permeation compared to PG and MBGNP groups when analyzed by dentinal tubule permeation tests. In the application of dentinal tubule occlusion, 30% phosphate MBG is the most recommended group. 
